# Discriminative Ability of the Charlson Comorbidity Index for Long-Term Mortality in a General Population: Nationwide, Population-Based Study of 10 Million Adults in Sweden

**DOI:** 10.2147/CLEP.S568699

**Published:** 2025-11-26

**Authors:** Marcus Westerberg, Hans Garmo, Jonas F Ludvigsson, Pär Stattin, Rolf Gedeborg

**Affiliations:** 1 Department of Surgical Sciences, Uppsala University, Uppsala, Sweden; 2 Department of Medical Epidemiology and Biostatistics, Karolinska Institutet, Solna, Sweden; 3 Department of Pediatrics, Örebro University Hospital, Örebro, Sweden; 4 Department of Medicine, Columbia University College of Physicians and Surgeons, New York, NY, USA

**Keywords:** Charlson Comorbidity Index, comorbidity, survival, discrimination, validation

## Abstract

**Background:**

The Charlson Comorbidity Index (CCI) is widely used to control confounding factors in epidemiological studies. There is a need to characterize the performance of CCI in a contemporary general population. This study assessed the discriminative ability of CCI for long-term mortality in relation to age, sex, data source, length of look-back period, and calendar time.

**Methods:**

Open cohort study of all adults residing in Sweden for at least one year between 2003 and 2022. Multiple versions of the CCI were calculated based on 1–10 years of lookback in the Swedish National Patient Register. Discrimination was determined using the concordance index (C-index). Kaplan-Meier curves were used to describe 10-year survival.

**Results:**

About 10395689 unique individuals had a median follow-up for mortality of 10 years. The C-index of CCI increased with the length of the look-back period, from 0.694 (95% confidence interval [CI], 0.694–0.694) at 1 year, to 0.784 (95% CI, 0.783–0.784) at 5 years, and to 0.808 (95% CI, 0.807–0.808) at 10 years. Discrimination was highest in subjects aged 60 years, for whom the discrimination of CCI_10years_ was higher in women (C-index, 0.794 [95% CI, 0.788–0.799]) than in men (C-index, 0.730 [95% CI, 0.725–0.735]). Within age strata above 70 years, the risk of death was much lower in subjects with CCI = 0 vs 1, 2 vs 3, and 3 vs ≥4, but not in those with CCI = 1 vs 2.

**Conclusion:**

The CCI discriminated risk of death best using a look-back period of at least 5 years and performed better in women than in men in most age groups. Our findings offer insights into both the practical utility of the CCI and the interpretation of studies in which it has been applied.

## Plain Language Summary

We assessed the discriminative ability of the Charlson Comorbidity Index (CCI) for long-term mortality in relation to age, sex, data source, length of look-back period, and calendar time in a general adult population representative of all types of hospital and specialist care. Discrimination was best using a look-back period of at least five years, varied strongly with age, decreased notably in older age groups, and was higher in women than in men in most age groups. This comprehensive assessment of the discriminatory ability of the CCI supports the validity of results from studies that use the CCI and offers insights into the practical utility of the CCI, including decisions regarding its implementation, such as the choice of lookback period and source of underlying data.

## Introduction

The widely used Charlson Comorbidity Index (CCI) was originally developed to predict risk of death from chronic diseases in observational studies and is frequently used in register-based research to describe comorbidity and to control for confounding from comorbidity.^1^ The CCI has been extensively validated in different disease conditions and healthcare settings.^2^ The discriminatory ability of the CCI may vary according to patient characteristics such as age, sex, and the specific primary disease or condition studied, but also across countries and populations and over calendar time due to differences in health care setting and rating methods.^2^ Furthermore, coding practices and the chosen length of the look-back period for diagnoses can influence the predictive ability of CCI. ICD coding practices vary widely across countries and even within regions of the same country.^3–5^ Differences include the number of allowable diagnostic fields, definitions of the main condition, mandatory data fields, and the use of national modifications of ICD.^6^ Longer look-back periods often lead to increased accuracy.^2^ For example, a look-back of 10 years was required for stable estimation of multimorbidity prevalence and health outcomes prediction,^7^ and a look-back period of 6 years before diagnosis was found to be optimal in another study of patients with laryngeal cancer.^8^

A comprehensive assessment of the discriminatory ability of the CCI in a general adult population representative of all types of hospital and specialist care in an entire country would support the validity of the results of studies that use the CCI. It can also inform the choice of look-back and data source and provide insights on its potential strengths and weaknesses in terms of its ability to predict mortality in different subgroups and its usefulness as a proxy for overall health status.

The aim of this nationwide, population-based cohort study was to describe the ability of the CCI to discriminate risk of death from any cause according to the type of data source used, length of the look-back period, length of follow-up, and calendar time. We also aimed to determine the influence of age and sex on the discriminative ability of the CCI and to describe the ability of CCI to identify differences in survival probability within strata of age and sex.

## Materials and Methods

### Study Design and Setting

The study used an open cohort design with inclusion between January 1, 2003, and December 31, 2022.^9^ All adults (≥18 years) who had resided in Sweden for at least one year on January 1, 2003, were included. On January 1 each subsequent year new subjects were added if they fulfilled one of two criteria: (a) had reached the age of 18 and had resided in Sweden for at least one year, or (b) had migrated to Sweden after January 1, 2003, were ≥18 years old, and had resided in Sweden for at least one year.

The Swedish Ethical Review Authority approved the study [2023–04700-01] and waived the need for informed consent due to its registry-based nature.^10^ Individual-level data from the different registers were linked by use of the unique Swedish Personal Identity Number.^11^ All data was pseudonymised by Statistics Sweden prior to delivery to the researchers. We followed TRIPOD (Transparent Reporting of a multivariable prediction model for Individual Prognosis Or Diagnosis) guidelines for reporting (Supplement Table S1).^12^

### Data Sources

Demographic data to define the study cohort was obtained from the Total Population Register held at Statistics Sweden.^13^ The National Patient Register held by the Swedish National Board of Health and Welfare contains information on all in-hospital care and out-patient specialist care in Sweden with a nation-wide coverage of in-patient care since 1987 and specialist out-patient care since 2001.^14^,^15^ During the study period diagnoses from the types of healthcare encounters included in The National Patient Register were recorded according to the Swedish modifications of ICD-9 in 1987–1997 and of ICD-10 (ICD-10-SE) from 1997 and onwards. Transition from ICD-9 to ICD-10 was complete from 1998. ICD-10-SE has few modifications compared to the original ICD-10 version. Validation studies indicate that coding accuracy is diagnosis-specific.^16–18^ Data was available up to 10 years prior to inclusion in the study (at the earliest from the age of 18) and onwards.

The date of death was extracted from The Cause of Death Register^19^ until December 31, 2023.

### Calculation of the Charlson Comorbidity Index

We used an adaption of the CCI for register-based research in Sweden to map main and secondary diagnoses in The National Patient Register to the original CCI disease condition categories.^20^ It is based on ICD-9 for diagnoses in 1987–1998 and ICD-10 for diagnoses from 1997. The CCI is often computed using the history of diagnoses registered during a chosen number of years backward in time (called *look-back period*) relative to a specific date. In this study, ten different versions of the CCI (CCI_1year_-CCI_10years_) were calculated based on 1–10 years of look-back period for discharge diagnoses from both in-hospital care and out-patient specialist care registered in The National Patient Register. In three separate analyses, we computed the CCI using (1) only in-hospital stays, (2) by requiring the same diagnosis (ICD-code) to appear either in at least one in-hospital stay, and/or in at least two separate out-patient visits, during the look-back period, and (3) by requiring the same diagnosis to appear either at least once as main diagnosis, and/or as secondary diagnosis on at least two separate dates, during the look-back period.

### Statistical Analyses

#### Pseudo-Individuals and Censoring

Since individuals could be included in the cohort at different dates and to obtain an even distribution of index dates across calendar time, we generated for each individual an index date each quarter from the first quarter of the year of inclusion in the cohort. Each individual could consequently have up to 80 different index dates. We considered each combination of an index date and an individual as a *pseudo-individual* (see Supplementary Methods for details). Only pseudo-individuals that were alive and had resided in Sweden for at least one year up to and including the index date were included. Each version of the CCI was computed at every index date. In-hospital stays that had not ended before the index date were not included in these computations.

Follow-up started at the index date and pseudo-individuals were censored at the date of migration (after the index date, if any) or on December 31, 2023. The length of follow-up was calculated using the reverse Kaplan-Meier method.^21^ For each pseudo-individual, a health care encounter was defined as a unique date of admission to in-hospital care or out-patient specialist care visit registered in The National Patient Register within a defined look-back period.

#### Discrimination

Analyses were performed using R version 4.0.2. The discriminatory performance of the CCI was assessed using the concordance index (C-index).^22^ A C-index of 0.5 for the CCI means that the CCI is unrelated to the risk of death, and a C-index of 1 means that it perfectly aligns with risk of death. The C-index for CCI using 1 year of look-back period can for example be 0.726 when assessing 1-year mortality in hospitalized older adults.^23^ We computed the C-index after 1 and 5 years of follow-up along with 95% confidence intervals (CIs). We used a robust variance estimator to account for the dependence between multiple pseudo-individuals for each individual when estimating the variance (see Supplementary Methods for details). Analyses were performed on all individuals and in subgroups defined by age, sex, and calendar time.

#### Survival

We also assessed 10-year survival by use of Kaplan-Meier curves with corresponding 95% confidence intervals using the pseudo-individuals.

## Results

### Baseline Characteristics

There were 10,395,742 unique subjects in the study cohort and each subject contributed with between 1 and 80 unique index dates and an average of 58 unique index dates, generating a total of 599,679,739 pseudo-individuals (Table 1). The median age was 49 years (interquartile range [IQR] 34–64 years) and the sex distribution was overall balanced.

**Table 1:** Baseline Characteristics for 10,395,689 Unique Adult Subjects Assessed as 599,436,406 Pseudo-Individuals

	All Ages		18-49 Years		50-69 Years		≥70 Years	
	N	(%)	N	(%)	N	(%)	N	(%)
**Pseudo-individuals (N; x100 000)**	600	(100)	307	(100)	186	(100)	107	(100)
**Sex**								
Men	2969	(49.5)	1570	(51.1)	932	(50.1)	468	(43.7)
Women	3028	(50.5)	1500	(48.9)	926	(49.9)	601	(56.3)
Number of healthcare encounters* during **the previous year**								
0	3502	(58.4)	1989	(64.8)	1065	(57.3)	447	(41.9)
1-3	1798	(30)	829	(27)	569	(30.6)	401	(37.5)
≥4	696	(11.6)	252	(8.2)	224	(12)	221	(20.7)
Number of healthcare encounters* during **the previous 10 years**								
0	908	(15.1)	562	(18.3)	278	(14.9)	68	(6.3)
1-3	1557	(26)	925	(30.1)	475	(25.6)	157	(14.7)
≥4	3532	(58.9)	1582	(51.5)	1105	(59.5)	845	(79)
**Charlson comorbidity index** (1-year look-back period)								
0	5554	(92.6)	2997	(97.6)	1703	(91.6)	854	(79.9)
1	204	(3.4)	42	(1.4)	71	(3.8)	90	(8.5)
2	163	(2.7)	25	(0.8)	61	(3.3)	78	(7.3)
3	32	(0.5)	2	(0.1)	9	(0.5)	21	(2)
≥4	44	(0.7)	5	(0.2)	14	(0.8)	25	(2.3)
**Charlson comorbidity index** (10-year look-back period)								
0	4206	(70.1)	2440	(79.5)	1276	(68.7)	490	(45.9)
1	425	(7.1)	118	(3.8)	146	(7.9)	161	(15.1)
2	283	(4.7)	43	(1.4)	105	(5.7)	134	(12.5)
3	90	(1.5)	6	(0.2)	26	(1.4)	59	(5.5)
≥4	104	(1.7)	8	(0.3)	29	(1.6)	67	(6.3)
**Occurrence of ICD-10 code chapter during** **the previous 10 years**								
**A or B** (infectious and parasitic diseases)	618	(10.3)	314	(10.2)	156	(8.4)	148	(13.9)
**C or D** (neoplasms and diseases of the blood and blood-forming organs)	1092	(18.2)	338	(11)	381	(20.5)	373	(34.9)
**E** (endocrine, nutritional, and metabolic diseases)	697	(11.6)	188	(6.1)	235	(12.7)	274	(25.6)
**F** (mental and behavioral disorders)	677	(11.3)	366	(11.9)	191	(10.3)	121	(11.3)
**G** (diseases of the nervous system)	593	(9.9)	192	(6.3)	218	(11.7)	183	(17.1)
**H** (ear, nose, or throat diseases)	1366	(22.8)	354	(11.5)	455	(24.5)	557	(52.1)
**I** (diseases of the circulatory system)	1112	(18.5)	158	(5.1)	406	(21.8)	548	(51.3)
**J** (diseases of the respiratory system)	777	(13)	353	(11.5)	218	(11.7)	207	(19.3)
**K** (diseases of the digestive system)	1129	(18.8)	413	(13.5)	387	(20.8)	328	(30.7)
**L** (diseases of the skin and subcutaneous tissue)	935	(15.6)	387	(12.6)	297	(16)	251	(23.5)
**M** (diseases of the musculoskeletal system and connective tissue)	1541	(25.7)	551	(17.9)	573	(30.9)	417	(39)
**N** (diseases of the genitourinary system)	1439	(24)	640	(20.8)	452	(24.3)	347	(32.5)
**R** (symptoms, signs, and abnormal clinical and laboratory findings, not elsewhere classified)	2043	(34.1)	850	(27.7)	653	(35.1)	540	(50.6)
**S or T** (injury, poisoning and certain other consequences of external causes)	1841	(30.7)	937	(30.5)	523	(28.1)	381	(35.6)
**Z** (factors influencing health status and contact with health services)	2745	(45.8)	1284	(41.8)	795	(42.8)	666	(62.3)
**O, P, Q, U or Y** (miscellaneous)	706	(11.8)	623	(20.3)	47	(2.5)	36	(3.4)

### Frequency of Health Care Encounters

**Figure 1:**
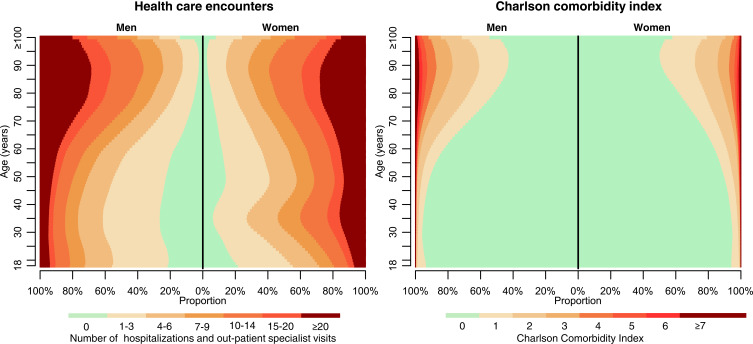
Number of hospitalizations and Charlson Comorbidity Index (CCI). *Left panel*: The number of hospitalizations and specialist out-patient visits in 10395689 unique adult subjects assessed as 599436406 pseudo-individuals. *Right panel*: The CCI computed using ICD-codes from hospitalizations and specialist out-patient visits during a 10-year look-back period. Results are presented by sex.

The frequency of healthcare encounters increased with increasing age, but also in the age group 18–49 years at least one healthcare encounter was registered in 35% during the year preceding the index date and in 82% during the 10 years preceding the index date (Table 1, Figure 1). The number of hospitalizations and specialist out-patient visits was higher in women (median 6; IQR: 2–15) than in men (median 4; IQR: 1–10) during the 10 years preceding the index date (Supplementary Table S2), and this difference was more pronounced in pseudo-individuals aged around 30 years. The mean number of ICD-10 codes per healthcare encounter in unique individuals increased with about 0.02 codes per year from 1.43 in 2003 to 1.81 in 2022. There were substantial differences between age groups in the distribution of some, but not all, diagnosis categories (Table 1).

Of all 6.15 billion registered healthcare encounters within the previous 10 years of all pseudo-individuals’ index dates, 12% were in-hospital stays and this proportion increased with age from 7% in 18-year old subjects to 12% in those 70 year old and 28% in those 100 years or older.

### The Charlson Comorbidity Index

The CCI increased with age and increasing length of the look-back period (Table 1, Figure 1). In those aged 18–49 years 80% had a CCI_10years_ = 0, compared to 69% in those 50–69 years old. Using a 1-year look-back period 93% had CCI_1year_ = 0, compared to 70% using a 10-year look-back. A similar pattern was seen across the age range. Men had somewhat higher CCI than women in most age groups (Supplementary Table S1, Figure 1).

### Discrimination – Look-Back Period and Calendar Time

**Figure 2:**
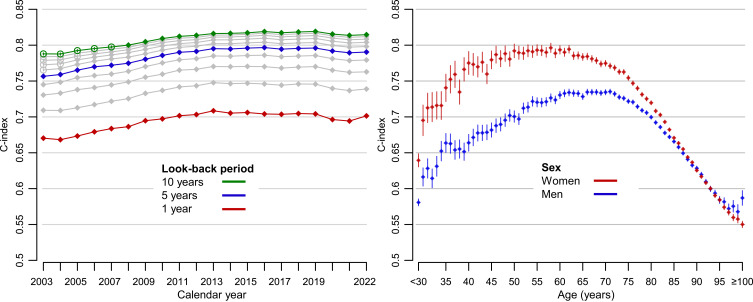
Discriminatory ability (C-index) of the Charlson Comorbidity Index (CCI). C-index was computed using 1 year of follow-up for mortality. *Left panel*: Comparison of look-back periods for diagnosis codes ranging between 1–10 years. Grey lines indicate the C-index of the CCI using look-back periods from 2–4 and 6–9 years, and filled circles indicate that the corresponding CCI was computed entirely based on ICD-10 codes whereas empty circles indicate that the calculation used both ICD-9 and ICD-10 codes. *Right panel*: Comparison of how discrimination depends on age and sex using a 10 year of look-back period. Pseudo-individuals aged <30 years were analysed as one group since there were few events in this age span.

The discriminative ability of the CCI for 1-year mortality increased with increasing length of the look-back period (Figure 2). The increase in C-index was larger going from a 1-year look-back (C-index 0.694 [95% CI, 0.694 −0.694]) to a 5-year look-back (C-index 0.784 [95% CI, 0.783–0.784]), compared to going from a 5-year to a 10-year look-back (C-index 0.808 [95% CI, 0.807–0.808]).

Discrimination was quite stable over calendar time. If anything, there was a slight increase in CCI particularly between 2003 and 2012 (Figure 2). Eg the C-index of CCI_10years_ increased from 0.788 (95% CI, 0.787–0.789) in 2003 to 0.814 (95% CI, 0.812–0.815) in 2012 and to 0.815 (95% CI, 0.813–0.816) in 2022.

### Discrimination – Age and Sex

Discrimination of the CCI varied according to both age and sex, with a peak around age 50–60 in women and age 60–70 in men and thereafter decreased with increasing age in both sexes (Figure 2). Although discrimination was overall somewhat lower in women (C-index of CCI_10years_, 0.802; 95% CI, 0.802–0.802) than in men (C-index of CCI_10years_, 0.814; 95% CI, 0.813–0.814), discrimination within age strata up to 85 years was often much higher in women. For example, at age 60 the C-index of CCI_10years_ was 0.794 (95% CI, 0.788–0.799) in women and 0.730 (95% CI, 0.725–0.735) in men. The difference between women and men decreased in older age groups.

### Discrimination – Data Source and Length of Follow-Up

Discrimination was somewhat lower when only diagnoses from hospitalizations were included in the computation of the CCI for all look-back periods, eg C-index 0.656 (95% CI, 0.655–0.65) for CCI_1year_ and C-index 0.790 (95% CI, 0.789–0.790) for CCI_10years_, in both men and women and in most ages (Supplementary Figure S1). Discrimination was also slightly lower when the same diagnosis had to appear either in at least one in-hospital stay, and/or in at least two separate out-patient visits, and somewhat lower when the same diagnosis had to appear either at least once as main diagnosis, and/or as secondary diagnosis on at least two separate dates.

The median duration of follow-up was 10 years (IQR: 5–15 years) and 13% of the pseudo-individuals died during follow-up (specifically 50% of subjects aged at least 70 years) (Supplementary Table S3). Although discrimination was somewhat lower for all look-back periods when using 5 years of follow-up, eg C-index 0.643 (95% CI, 0.643–0.643) for CCI_1year_ and C-index 0.756 (95% CI, 0.755–0.756) for CCI_10years_, the patterns in sex and age were similar to when using 1 year of follow-up (Supplementary Figure S2).

### Risk of Death

**Figure 3:**
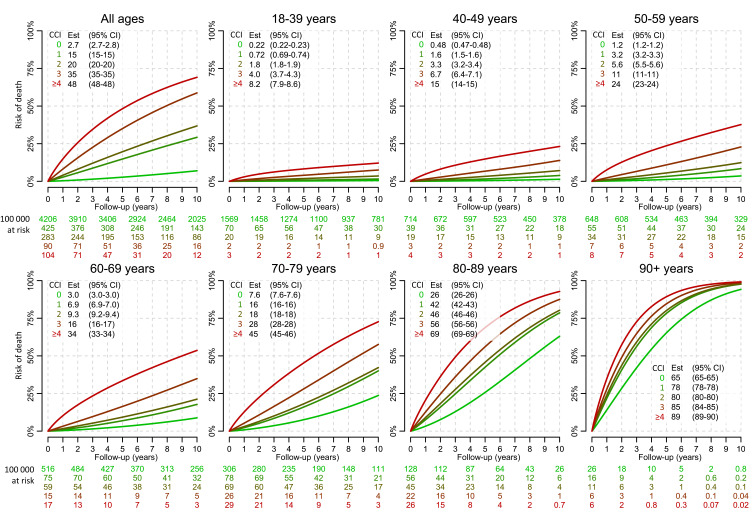
Risk of death according to age and the Charlson Comorbidity Index (CCI). The CCI was computed using a 10-year look-back period. The estimates of risk of death after 5 years of follow-up are provided with confidence intervals (CIs) in the corresponding boxes. Numbers at risk are provided for each level of CCI per 100000 pseudo-individuals.

There was a wide range in risk of death according to age and CCI. For example, risk of death within 5 years for pseudo-individuals with CCI_10years_ = 0 was 2.7% (95% CI, 2.7–2.8%), and increased with increasing CCI to 48% (95% CI, 48–48%) for those with CCI_10years_ ≥ 4 (Figure 3). The difference increased with age, except in pseudo-individuals aged 90 years or above for whom survival was more similar between the CCI levels. The risk of death in pseudo-individuals with CCI_10years_ = 1 was similar to those with CCI_10years_ = 2, especially in those aged 70 years or above (Figure 3, Supplementary Figure S3). For example, risk of death within 5 years in those aged 70 to 79 years with CCI_10years_ = 1 was 16% (95% CI, 16–16%) and 18% (95% CI, 18–18%) in those with CCI_10years_ = 2. Similar patterns were observed when stratifying by sex (Supplementary Figures S4 and S5) and for CCI based on shorter look-back periods (data not shown).

## Discussion

### Summary of Findings

In this nationwide population-based cohort study of more than 10 million individuals, the CCI discriminated risk of death best using a look-back period of at least 5 years with data from in-hospital care and specialized out-patient care. The overall discrimination slightly improved between 2003 and 2012.

Discrimination varied strongly with age and decreased notably in older age groups. Overall, discrimination was somewhat lower in women than in men, but when stratified for age the discrimination was instead clearly higher in women than in men, up to age 85. The CCI identified groups with distinctively different risk of death within strata of age, although individuals with CCI = 1 and 2 aged 70 or above had similar survival probability within strata of age.

### Interpretation and Previous Studies

In a recent evaluation comparing different look-back periods, 10 years was required for stable estimation of multimorbidity prevalence and health outcomes prediction, which is in line with our study.^7^ In another study of patients with laryngeal cancer a look-back period of 6 years before diagnosis was found to optimal.^8^ For most purposes, a look-back between 5 and 10 years is superior to shorter look-back periods.

Discrimination was generally somewhat lower when only hospitalizations were used to compute the CCI, which suggest that there is overall some added value of the addition of specialized out-patient care encounters but the impact is not strong. In certain patient populations, the information from out-patient visits could be of greater importance.

We found a large variation in the discriminative ability of the CCI related to age, with a peak around 60 years. The reasons for a substantially lower discriminatory ability of the CCI in younger individuals is not clear. Younger subjects had fewer health care encounters than older, however, 82% of subjects aged 18–49 still had at least one healthcare encounter in the previous 10 years, which was comparable to the proportion in those aged 50–69. There was, however, notable differences in specific disease categories. An example is cardiovascular disease, which was uncommon in younger individuals but substantially more common with increasing age. In adolescents and younger adults traumatic injuries is the most common cause of death, but the CCI only captures chronic diseases and does not include diagnoses related to injury. While comorbidity has an impact on mortality from trauma,^24^ we speculate that comorbidity affects all-cause mortality less in younger compared to older individuals.

The discriminative ability was lower in older compared to younger subjects. Despite that older subjects had higher prevalence of healthcare encounters, higher prevalence of important comorbidities, and higher CCI, the ability of the CCI to predict mortality was lower. We speculate that frailty is less well reflected by diagnoses from previous hospitalizations and out-patient specialist care visits in the older age groups. In a previous study of 629 patients ≥65 years who underwent percutaneous coronary interventions, 66% of patients were frail or had intermediate frailty according to the Fried and Walston criteria.^24^,^25^ Combined with the CCI, both the physical component of Short Form and the frailty assessment were independent risk factors for long-term mortality. This may in part explain the lower discrimination by the CCI in subjects above age 80 in our study.

Women had more health care encounters than men within each age group, and in particular around age 30, at this age this was likely due to pregnancy-related health care. Overall discrimination of the CCI was lower in women than in men, but clearly higher in women within each age stratum below 85 years. Although the difference in health care encounters did not translate to a higher CCI in women, it can at least partly explain why discriminatory ability of the CCI was higher in women than in men in specific age groups.

All versions of CCI in our study improved somewhat between 2003 and 2012 and performance was stable thereafter. This suggests that increased healthcare utilization and improved coding practices over time affected the CCI, since the transition from ICD-9 to ICD-10 was completed in Sweden already before 1998.^14^ Another potential reason is that over time in Sweden, the reimbursement to healthcare providers has increasingly been linked to registered diagnoses.

The CCI was able to separate groups with distinctly different survival probability when comparing CCI 0 vs 1 and CCI 2 vs 3 and 3 vs ≥4, also within strata of age and sex. There were, however, only small differences in survival in subjects with CCI 1 and 2 across all ages. Since most individuals with CCI > 0 have CCI 1 or 2, there is potential for improved CCI performance in individuals with one or two diagnoses of disease conditions corresponding to 1 point (eg, myocardial infarction and congestive heart failure) and/or 2 points (eg, hemiplegia and malignancy).

### Strengths and Limitations

Strengths of our study include that it was based on a nationwide, population-based cohort in a largely tax-funded healthcare system accessible to all permanent residents and that The National Patient Register has a nationwide coverage of inpatient care since 1987 and specialist outpatient care since 2001.^14^

This study also has some limitations. The National Patient Register does not include data from primary care, so failure to completely capture type 2 diabetes mellitus that is often managed in primary care is likely to occur. Previous validation studies in The National Patient Register indicate that coding accuracy is diagnosis-specific,^16–18^ which can influence the ability of the CCI to predict mortality. Other errors in registration such as erroneous dates or ICD-codes may also affect the quality of the CCI but we expect this to be of less concern.^16–18^

Our study was based on the entire Swedish population. While it is a strength for the study that it is very large and population-based, covering all types of medical conditions, the characteristics of this population, the healthcare system, and coding practices may not be fully applicable to other countries and healthcare settings.

### Implications and Future Work

Our results support extension of the look-back period to 5–10 years and use of diagnoses from both in-patient and specialist out-patient settings appearing at least once as main and/or secondary diagnosis in either of the sources to optimise mortality prediction. The extent to which this also translates into better control for confounding likely varies depending on the specific exposure-outcome association studied and the age-bracket in focus.

There may be opportunities to improve the performance of the CCI. Particularly in subjects above 80 years of age and in men (all ages). Improvements could likely be made by deriving new weights for CCI categories because of temporal changes in therapeutic strategies and prognosis, removing some codes that may be less relevant today (eg AIDS), requiring two or more code occurrences to increase specificity of identified disease conditions, and use separate weights for men and women.

A fundamental weakness of the CCI is that it is based on a set of pre-specified diagnoses. There is an opportunity to optimise measures of comorbidity by integrating weights from a larger set of diagnoses^26^ and include other types of healthcare data, such as prescribed medications^27^ and medical procedures, in a data-driven approach.^28^ This could improve discrimination of mortality and the ability to control for confounding.^29^

## Conclusions

In this nationwide population-based study of 10 million adults in Sweden, the discriminatory ability of the Charlson Comorbidity Index (CCI) for long-term mortality was highest when using a look-back period of at least 5 years. CCI varied strongly according to age and sex and clearly separated groups with different survival probability also within strata according to age and sex. Our findings provide valuable insights into both the practical utility of the Charlson Comorbidity Index—including decisions regarding its implementation, such as the choice of lookback period and source of underlying data—and the interpretation of studies in which it has been applied.

## Data Availability

The data cannot be shared publicly because the individual-level data contain potentially identifying and sensitive patient information and cannot be published due to legislation and ethical approval (https://etikprovningsmyndigheten.se). Use of the data from national health-data registers is further restricted by the Swedish Board of Health and Welfare (https://www.socialstyrelsen.se/en/) and Statistics Sweden (https://www.scb.se/en/) which are Government Agencies providing access to the linked healthcare registers.
